# Comparison of collagen proportionate areas in liver fibrosis quantification between chronic hepatitis B and C

**DOI:** 10.1097/MD.0000000000004736

**Published:** 2016-09-02

**Authors:** Sheng-Hung Chen, Cheng-Yuan Peng, I-Ping Chiang, Hsueh-Chou Lai, Chiung-Ju Lee, Wen-Pang Su, Jung-Ta Kao, Po-Heng Chuang

**Affiliations:** a Graduate Institute of Clinical Medical Science; b School of Medicine; c College of Chinese Medicine, China Medical University; d Division of Hepatogastroenterology, Department of Internal Medicine; e Department of Pathology, China Medical University Hospital, Taichung, Taiwan.

**Keywords:** acoustic radiation force impulse, chronic hepatitis B, chronic hepatitis C, collagen proportionate area, liver fibrosis

## Abstract

Few studies have compared the distinct hepatic collagen morphometrics of chronic hepatitis B (CHB) and chronic hepatitis C (CHC). This study compared the discrepancies between CHB and CHC in liver fibrosis (F) quantification by using the collagen proportionate area (CPA) and liver stiffness (LS) measured with shear wave velocity (SWV).

This study enrolled 274 eligible consecutive patients diagnosed with CHB (n = 137) or CHC (n = 137). Their ages ranged from 20 to 80 years (median = 50). In total, 154 patients (56.2%) were male. Participant LS was measured by using acoustic radiation force impulse elastography preceding an immediate percutaneous liver biopsy. The total proportion of the collagen stained with picrosirius red to the total tissue area was expressed as the CPA percentage, which was stratified into portal–bridging (PB) and perisinusoidal (PS) proportionate areas (PAs).

Based on the METAVIR F staging system, 36 (26.3%), 36 (26.3%), 28 (20.4%), and 37 (27.0%) participants in the CHB group and 34 (24.8%), 45 (32.9%), 34 (24.8%), and 24 (17.5%) participants in the CHC group were staged as F1, F2, F3, and F4, respectively. Both the total CPAs and PBPAs were significantly (*P* < 0.05) higher in the CHC group than in the CHB group within all F-stratified subgroups. The SWVs were significantly (*P < *0.05) higher in the CHC group than in the CHB group only within the F2, F3, and F4 subgroups. However, the PSPAs did not differ significantly between the CHC and CHB groups within all subgroups. Multiple regression analysis revealed that viral hepatitis etiology (*P* < 0.001), METAVIR F stages (*P* < 0.001), and platelet count (*P* = 0.007) were independent factors correlated with the CPA (*R*^2^ = 0.543, *P* < 0.001).

In conclusion, both the F stage-stratified CPAs and SWVs tended to be higher in cases of CHC than in those of CHB. The type of viral hepatitis significantly affected both the CPA and SWV values. The PBPAs were more closely correlated with F stages and SWV than were the PSPAs.

## Abstract 2

Supplemental Digital Content is available in the text

## 1 Introduction

Chronic hepatitis B (CHB) infection remains a major global healthcare challenge.^[1]^ High-level hepatitis B virus (HBV) replication is a major risk factor for disease progression to end-stage complications such as decompensated cirrhosis and hepatocellular carcinoma (HCC).^[2]^ However, permanent suppression of HBV replication is currently achievable and can lead to the reversal of fibrosis (F) and even regression from cirrhosis.^[3]^

Over the past decade, emerging molecular insights into the bidirectional model of liver fibrogenesis and potential treatment targets for F reversal have necessitated longitudinal noninvasive measures for evaluating progression or reversal of F.^[4]^

Despite the demand for internal and external validation, reports on liver stiffness measurement (LSM) using acoustic radiation force impulse (ARFI) have exhibited promising levels of precision and validity in liver F evaluation^[5]^; however, the conventional liver F staging has remained the gold standard test and few studies to date have examined the discrepancies between CHB and chronic hepatitis C (CHC).

The invasive nature of another modality, collagen proportionate area (CPA), may compromise its role as a universally practical, diagnostic, and prognostic tool for liver diseases. However, the CPA has several advantages in refining the hepatic F quantification for parenchymal F progressing from the portal area to extensive cirrhosis. The area proportion–based or pixel proportion–based CPA determined by picrosirius red staining through digital image analysis is highly correlated with conventional F staging,^[6–8]^ the hepatic vein pressure gradient,^[8–11]^ liver stiffness (LS),^[7,11]^ cirrhosis substages,^[10,12]^ and prognosis.^[8,13]^ The CPA is also significantly correlated with several liver reserve surrogates and serum marker models including Model for End-Stage Liver Disease scores, international normalized ratio of prothrombin time, and bilirubin levels.^[14,15]^ Therefore, the broad spectrum of CPA may serve as a promising discriminator in substaging cirrhosis to identify hepatic decompensation either at baseline^[14]^ or over time.^[8,16]^

Because of its distinct pathogenesis, the characteristics of liver fibrogenesis differ between CHB and CHC. However, few studies have compared the hepatic collagen morphometries of CHB and CHC or those of portal–bridging (PB) and perisinusoidal (PS) proportionate areas (PAs). ^[17,18,19]^ In addition, no study has directly compared the diagnostic performances of the CPA with those of LSM using ARFI when dichotomizing F stages in CHB.

Therefore, we aimed to implement direct comparisons of liver F quantifications in CHB by contrasting the CPA with LS and investigating the discrepancies between CHB and CHC in both the CPA and LS, with the total CPA being stratified into PBPA and PSPA.

## 2 Methods

### 2.1 Ethics statement

Written informed consent was obtained from all participants. The study protocol was approved by the Research Ethics Committee of China Medical University Hospital and was developed in accordance with the Declaration of Helsinki, 1975.

### 2.2 Patients

This study screened consecutive patients diagnosed with CHB or CHC at the medical center from January 2013 to January 2016. The patients were enrolled in a prospective cohort for the analysis of antiviral treatment responses. CHB infection was determined by positive results for serum HBV surface antigens (Abbott Laboratories, Abbott Park, IL) for more than 6 months. CHC infection was determined by positive results for hepatitis C virus (HCV) antibodies (Abbott Laboratories, Abbott Park, IL) for more than 6 months with detectable serum HCV RNA (detection limit: 15 IU/mL) (COBAS Ampliprep/COBAS TaqMan HCV test, Roche Diagnostics, Branchburg, NJ). The patient exclusion criteria comprised age <20 years, hepatitis B and C coinfection, human immunodeficiency virus coinfection, decompensated cirrhosis (a Child–Turcott–Pugh score value ≥7),^[20]^ HCC, primary biliary cirrhosis, primary sclerosing cholangitis, Wilson disease, autoimmune hepatitis, hemochromatosis, extrahepatic cholestasis, alcoholic liver disease, myeloproliferative disorders, thalassemia, cardiac congestion, blood product transfusion in the preceding 30 days, pregnancy, and serum creatinine higher than 221 μmol/L (2.5 mg/dL).

### 2.3 Blood tests

Complete blood count analyses (Sysmex, Hyogo, Japan) and blood biochemistry (Beckman Coulter, Brea, CA) were performed in the central laboratory of the medical center. The serum HBV DNA levels were measured using the Cobas Amplicor HBV monitor 2.0 (lower limit of detection, 12 IU/mL) (Roche Diagnostics, Branchburg, NJ). The HCV RNA was quantified at baseline. Aspartate transaminase-to-platelet ratio index = (aspartate transaminase/upper limit of normal, 34 IU/L)/platelet count (10^9^/L) × 100.

### 2.4 LSM using ARFI

The participants underwent percutaneous right-lobe liver biopsy within 1 hour of blood sampling and LSM after 3 hours of fasting.^[21]^

ARFI technology was integrated into the ultrasound system (Acuson S2000 with a Siemens 4C1 curved array, 4.00 MHz for B-mode, 2.67 MHz for push pulses, and 3.08 MHz for detection pulses; Siemens Medical Solutions, Mountain View, CA). LS was measured using detection pulses and presented as shear wave velocity (SWV) in meters per second (m/s).

A single hepatologist experienced in digestive system ultrasonography and blinded to participant data implemented the LSMs. Cases were deemed reliable when the interquartile range (IQR) was lower than 30% of the median of 10 successful LSMs and the successful LSM rate was higher than 60%. Other cases were defined as unreliable and were excluded.

### 2.5 METAVIR scoring

Senior hepatologists performed the percutaneous right-lobe liver biopsies. The specimens were stained using Masson trichrome, hematoxylin and eosin, and reticulin and interpreted by a single experienced pathologist blinded to the LSM results and patient data. Adequate specimens were defined as those at least 15 mm in length and containing at least 5 portal tracts.^[22]^ Liver F was staged as F0: no F; F1: portal F without septa; F2: portal F with a few septa; F3: numerous septa without cirrhosis; and F4: cirrhosis. Necroinflammatory activity (A) was graded as A0: no A; A1: mild; A2: moderate; and A3: severe.^[23]^ Steatosis (S) was graded using a system comprising S0: no S; S1: mild, 1% to 5% hepatocytes containing visible macrovesicular S; S2: moderate, 6% to 32%; S3: marked, 33% to 66%; and S4: severe, 67% to 100%.^[24]^

### 2.6 CPA

The total CPA was determined as reported in previous studies.^[9,25,26]^ Liver tissue sections 3 to 5 μm in thickness were stained using picrosirius red (Sigma-Aldrich, St. Louis, MO) and incubated for 1 hour. The slides were then rinsed in distilled water and washed in 0.5% (w/v) of acetic acid solution for 1 minute at room temperature. Finally, the sections were dehydrated in 2 changes of 100% alcohol for 5 minutes each and 2 changes of xylene for 5 minutes each. Images were captured using a digital camera (Canon EOS 650D, Canon, Tokyo, Japan) connected to a desktop computer system. The images, magnified 200×, were edited using the Adobe Photoshop CS6 software platform (Adobe Systems, San Jose, CA). On this platform, the interactive thresholdings were finalized through consensus between the hepatologists and the pathologist by reading consecutive thin-cut tissue sections stained with picrosirius red, Masson trichrome, hematoxylin and eosin, and reticulin, respectively.

**Figure 1:**
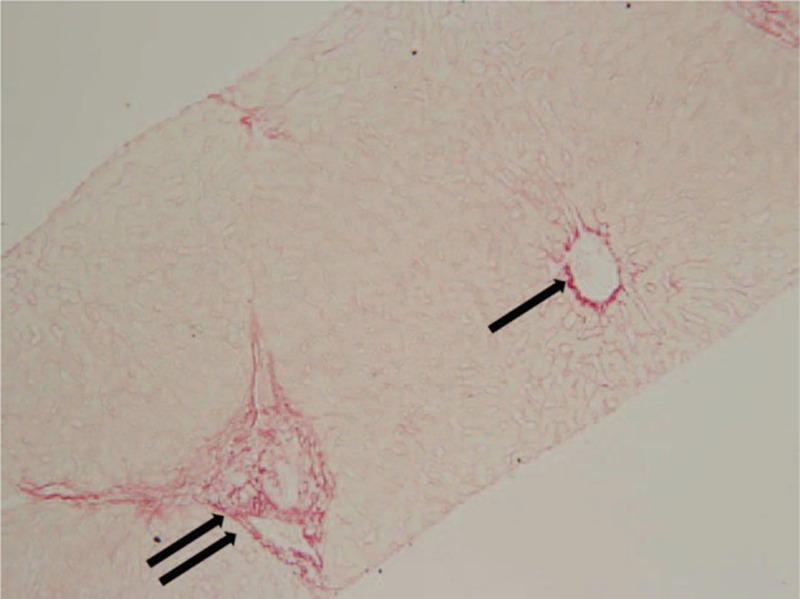
Portal–bridging (double arrow) and perisinusoidal (PS) (arrow) collagen proportionate areas in a 27-year-old male patient with chronic hepatitis B. METAVIR fibrosis stage = F1, total collagen proportionate area = 4.05%, shear wave velocity = 0.97 m/s, portal–bridging proportionate area = 2.15%, PS proportionate area = 1.90% (original magnification 200×).

The proportion of the numerator to denominator was formulated and calculated as a proportion of Σ fractals to Σ fractals. Both the numerator and denominator excluded the fractal areas of defects, artifacts, and lumens using Image-Pro Plus Version 7.0 (Media Cybernetics, Rockville, MD). Fractal areas of structural collagen irrelevant to the hepatitis disease process, including collagen in the walls of the portal tracts and central veins, were also subtracted from the numerator. An area proportion–based CPA percentage therefore represented the proportion of the total area of picrosirius red-stained collagen to the total tissue area. The total CPAs were stratified into PBPA and PSPA (Fig. 1).

### 2.7 Statistical analysis

Between-group and overall differences were estimated using the Mann–Whitney *U* test and Kruskal–Wallis test for continuous variables and the chi square test or Fisher exact test for proportions. Spearman rank correlation was used to evaluate the significance of correlations between 2 variables.

Receiver operating characteristic (ROC) analysis was employed to optimize the cutoff values in order to maximize the Youden index and evaluate diagnostic performances by using areas under the ROC curves (AUCs). The AUCs between the CPA and SWV were compared.^[27]^

The variables of age, sex, body mass index, comorbidities, METAVIR F stages, A grades, S grades, platelet count, international normalized ratio of prothrombin time, hemoglobin level, serum alanine transaminase (ALT), albumin, bilirubin, creatinine, and sodium levels were designated as covariates in the regression analyses.

Variables with a *P* value of less than 0.25 in univariate linear regression were included in the subsequent stepwise and multiple linear regression modeling. Data were analyzed using SPSS Version 17.0 for Microsoft Windows (SPSS, Chicago, IL). A 2-sided *P* value of <0.05 indicated statistical significance.

## 3 Results

### 3.1 Participants

In addition to the 137 patients with CHC,^[28]^ 155 patients diagnosed with CHB were screened after 4 cases were excluded because of unreliable LSMs. Moreover, patients diagnosed with alcoholic liver disease (n = 6), HCV coinfection (n = 6), and HCC (n = 6) were also excluded.

A cohort of 137 patients with CHB was incorporated into the study sample for comparing the CHB and CHC groups (Table 1). Of the 137 patients with CHB, 46 (33.6%) were women and 91 (66.4%) were men, with an age range of 20 to 77 years (median = 45 years). A total of 73 (53.3%) and 64 (46.7%) patients were HBeAg negative and positive, respectively.

**Table 1:**
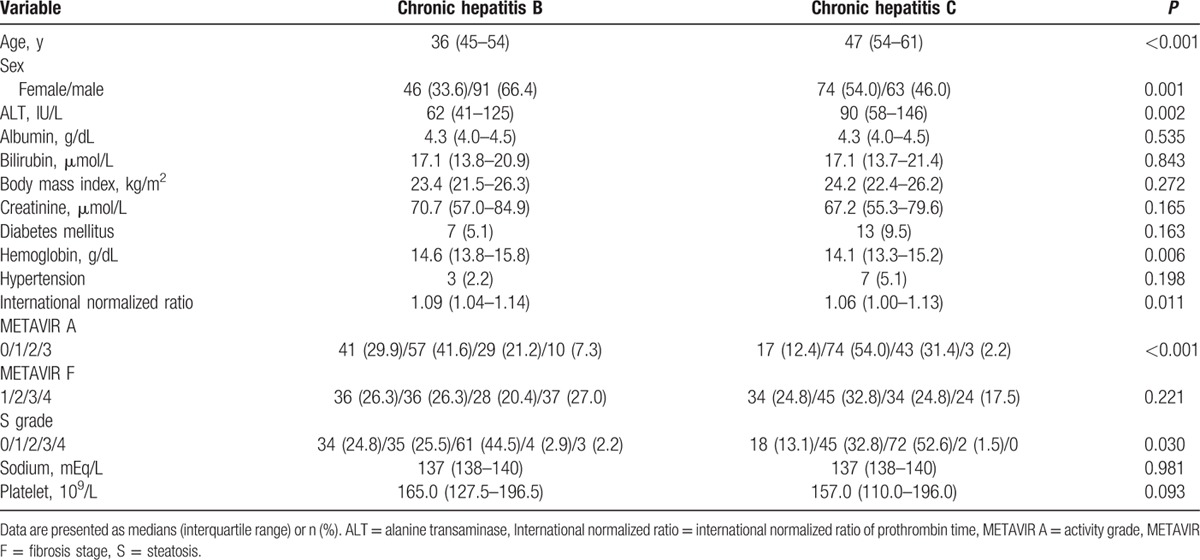
Patient characteristics.

Comparing the CHB (n = 137) and CHC (n = 137) groups, the age, percentage of female participants, ALT levels, distributions of METAVIR A grades, and S grades were significantly higher in the CHC group than in the CHB group. The levels of hemoglobin were significantly higher in the CHB than in the CHC group. There were no significant differences in METAVIR F stages or comorbidities including diabetes mellitus and hypertension.

### 3.2 Liver histology in CHB

On the basis of the METAVIR scoring system, 36 (26.3%), 36 (26.3%), 28 (20.4%), and 37 (27.0%) participants were staged as F1, F2, F3, and F4, respectively. Forty-one (29.9%), 57 (41.6%), 29 (21.2%), and 10 (7.3%) participants were graded as A0, A1, A2, and A3, respectively. According to S grading, 34 (24.8%), 35 (25.5%), 61 (44.5%), 4 (2.9%), and 3 (2.2%) participants were graded as S0, S1, S2, S3, and S4, respectively (Table 1).

### 3.3 CPA and SWV in CHB

**Figure 2:**
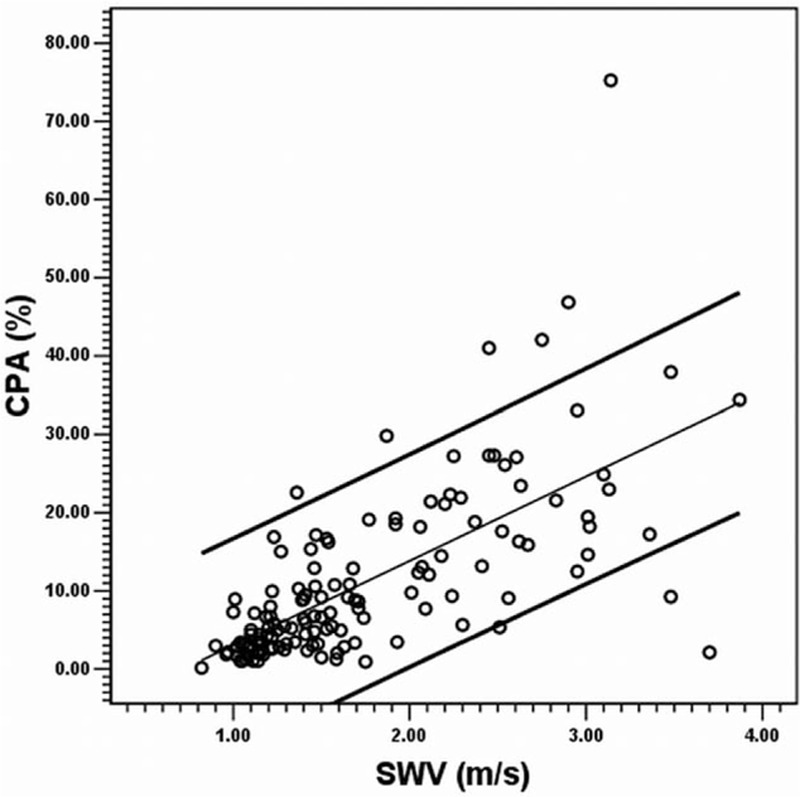
Scatter plot between the collagen proportionate area (*y* axis) and shear wave velocity (*x* axis) in the CHB group (n = 137). The CPA was predicted using SWV alone through univariate linear regression as a line of best fit (*R*^2^ = 0.459, *P* < 0.001) by using the formula: CPA (%) = −7.741 + SWV (m/s) × 10.793. Eight of the 137 cases (5.8%) exceeded the 95% confidence intervals. CHB = chronic hepatitis B, CPA = collagen proportionate area, SWV = shear wave velocity.

**Figure 3:**
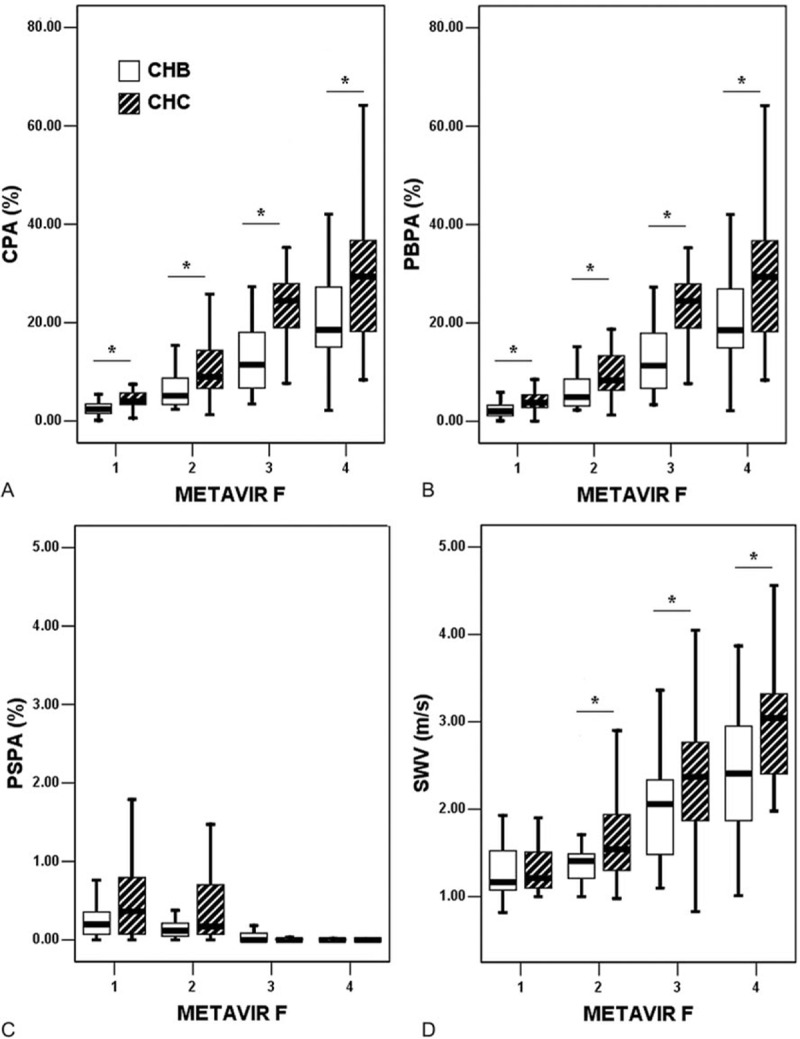
Box plots of the collagen proportionate areas (%) and shear wave velocity (m/s). Note that 36 (26.3%), 36 (26.3%), 28 (20.4%), and 37 (27.0%) cases in the CHB group and 34 (24.8%), 45 (32.9%), 34 (24.8%), and 24 (17.5%) cases in the CHC group were staged as F1, F2, F3, and F4, respectively. An asterisk indicates *P* < 0.05. CHB = chronic hepatitis B, CHC = chronic hepatitis C, CPA = collagen proportionate area, F = METAVIR fibrosis stage, PBPA = portal–bridging collagen proportionate area, PSPA = perisinusoidal collagen proportionate area, SWV = shear wave velocity.

In CHB, the total CPA could also be predicted using SWV alone through univariate linear regression as a line of best fit (*R*^2^ = 0.459, *P* < 0.001) by using the formula: CPA (%) = −7.741 + SWV (m/s) × 10.793. Eight of the 137 cases (5.8%) exceeded the 95% confidence intervals (Fig. 2). The medians and IQRs in each METAVIR F stage are shown in Fig. 3 for CPA, PBPA, PSPA, and SWV, respectively. In the CHB subgroup (n = 137), the CPAs (%, presented as the median and IQR in the parenthesis) were 2.41 (1.53–3.49) in F1 subgroup, 5.18 (3.34–8.78) in F2, 11.45 (6.67–18.42) in F3, and 18.51 (14.84–27.26) in F4, respectively. In the CHC subgroup (n = 137), the CPAs were 3.99 (3.36–5.85) in F1 subgroup, 9.03 (6.53–14.59) in F2, 24.48 (18.02–28.11) in F3, and 29.42 (17.78–36.85) in F4, respectively. The Spearman rank correlation coefficient between the CPA and METAVIR F stages was 0.798 (*P* < 0.001); between the PBPA and METAVIR F, the coefficient was 0.805 (*P* < 0.001); and between the PSPA and METAVIR F, it was −0.569 (*P* < 0.001). The Spearman rank correlation coefficient between the CPA and SWV was 0.721 (*P* < 0.001); between the PBPA and SWV, the coefficient was 0.720 (*P* < 0.001); and between the PSPA and SWV, it was −0.353 (*P* < 0.001).

In the CHC group,^[28]^ the Spearman rank correlation coefficient between the CPA and METAVIR F stages was 0.819 (*P* < 0.001); between the PBPA and METAVIR F, the coefficient was 0.817 (*P* < 0.001); and between the PSPA and METAVIR F, it was −0.618 (*P* < 0.001). The Spearman rank correlation coefficient between the CPA and SWV was 0.706 (*P* < 0.001); between the PBPA and SWV, the coefficient was 0.704 (*P* < 0.001); and between the PSPA and SWV, it was −0.521 (*P*  0.001).

### 3.4 Comparisons of the CHB and CHC groups

When the comparisons were made within each METAVIR F subgroup, the CPAs were significantly higher in the CHC group than in the CHB group within the F1 (*P* < 0.001), F2 (*P* < 0.001), F3 (*P* < 0.001), and F4 (*P* = 0.028) subgroups. Likewise, the PBPAs were significantly higher in the CHC group than in the CHB group within the F1 (*P* = 0.001), F2 (*P* = 0.002), F3 (*P* < 0.001), and F4 (*P* = 0.028) subgroups. The SWVs were significantly higher in the CHC group than in the CHB group only within the F2 (*P* = 0.036), F3 (*P* = 0.008), and F4 (*P* = 0.001) subgroups. However, the PSPAs did not differ significantly between the CHC and CHB groups within the F1, F2, F3, and F4 subgroups (Fig. 3). Because the CHB group was significantly younger than the CHC group (Table 1), we further compared the CPAs between the CHB and CHC subgroups within each METAVIR F subgroup after stratifying by an age cutoff of 50 years. In the 132 cases with age <50 years, the CPA values were higher in the CHC subgroup than in the CHB subgroup, except for the METAVIR F4 subgroup. In the 142 cases with age ≥50 years, the CPA values were similarly higher in the CHC subgroup than in the CHB subgroup in every F subgroup (refer to Figure, Supplemental Digital Content which illustrates the box plots for CPA stratified by age, F stages, and viral hepatitis etiologies).

### 3.5 Use of CPA and SWV for dichotomizing fibrosis stages in CHB

**Figure 4:**
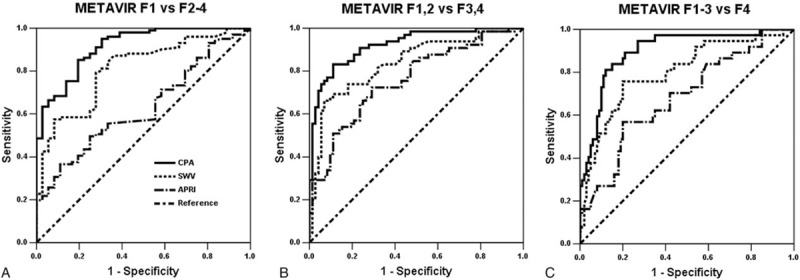
Receiver operating characteristic curves dichotomizing liver fibrosis stages in the CHB group (n = 137). To dichotomize (A) F1 versus F2 to F4, the areas under a receiver operating characteristic curve for the CPA was 0.914 (95% confidence interval: 0.864–0.965) and SWV was 0.810 (0.732–0.888) (CPA vs SWV, *P* = 0.029). For (B) F1 and F2 versus F3 and F4, the CPA was 0.921 (0.877–0.966) and the SWV was 0.836 (0.767–0.905) (*P* = 0.042). For (C) F1 to F3 versus F4, the CPA was 0.899 (0.840–0.958) and the SWV was 0.799 (0.712–0.886) (*P* = 0.060). APRI = aspartate transaminase-to-platelet ratio index, CHB = chronic hepatitis B, CPA = collagen proportionate area, F = METAVIR fibrosis stage, SWV = shear wave velocity.

To dichotomize METAVIR F stages using CPA (%) in CHB, the optimal cutoff values were 4.29 for F1 versus F2 to F4, 8.90 for F1 and F2 versus F3 and F4, and 13.12 for F1 to F3 versus F4. The optimal cutoff values of SWV (m/s) were 1.26 for F1 versus F2 to F4, 1.64 for F1 and F2 versus F3 and F4, and 1.82 for F1 to F3 versus F4. To dichotomize F1 versus F2 to F4, the AUCs for the CPA was 0.914 (95% confidence interval: 0.864–0.965) and SWV was 0.810 (0.732–0.888) (CPA vs SWV, *P* = 0.029). For F1 and F2 versus F3 and F4, the CPA was 0.921 (0.877–0.966) and the SWV was 0.836 (0.767–0.905) (*P* = 0.042). For F1 to F3 versus F4, the CPA was 0.899 (0.840–0.958) and the SWV was 0.799 (0.712–0.886) (*P* = 0.060) (Table 2 and Fig. 4).

**Table 2:**
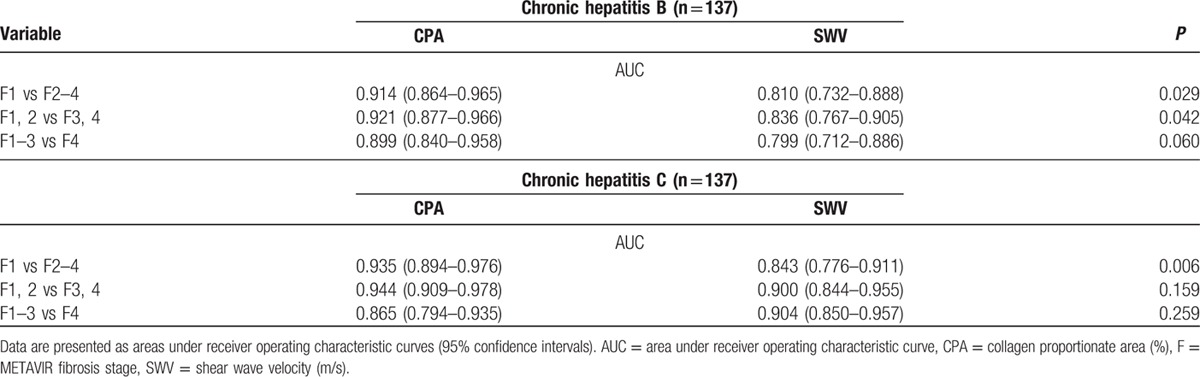
Comparisons in liver fibrosis dichotomization using the collagen proportionate area (%) versus the shear wave velocity (m/s).

### 3.6 Independent factors associated with CPA and SWV

During CPA modeling (*R*^2^ = 0.543, *P* < 0.001), the final multiple regression identified viral hepatitis etiology (CHC vs CHB) (*P* < 0.001), METAVIR F stages (vs F1) (*P* < 0.001), and platelet count (*P* = 0.007) as independently significant among all other covariates to correlate with the CPA (Table 3). Moreover, the final multiple regression (*R*^2^ = 0.527, *P* < 0.001) identified viral hepatitis etiology (CHC vs CHB) (*P* < 0.001), body mass index (*P* = 0.025), METAVIR A grades (vs A0) (*P* < 0.05), and METAVIR F stages (vs F1) as independently significant among all other covariates to correlate with the SWV (Table 4).

**Table 3:**
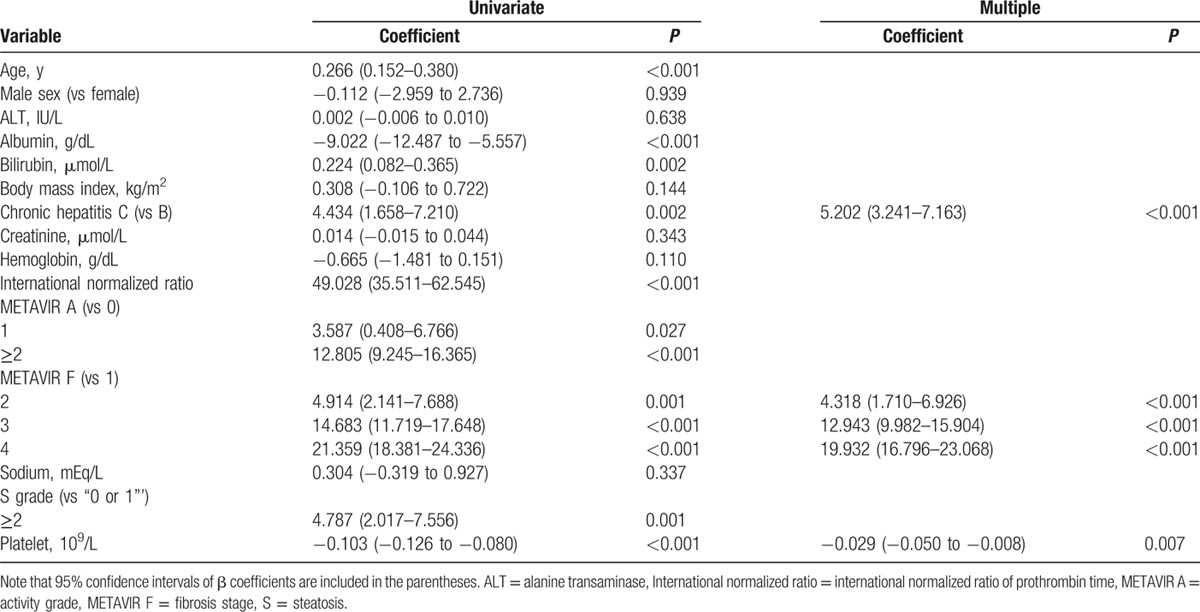
Multiple regression analyses for the collagen proportionate area (%).

**Table 4:**
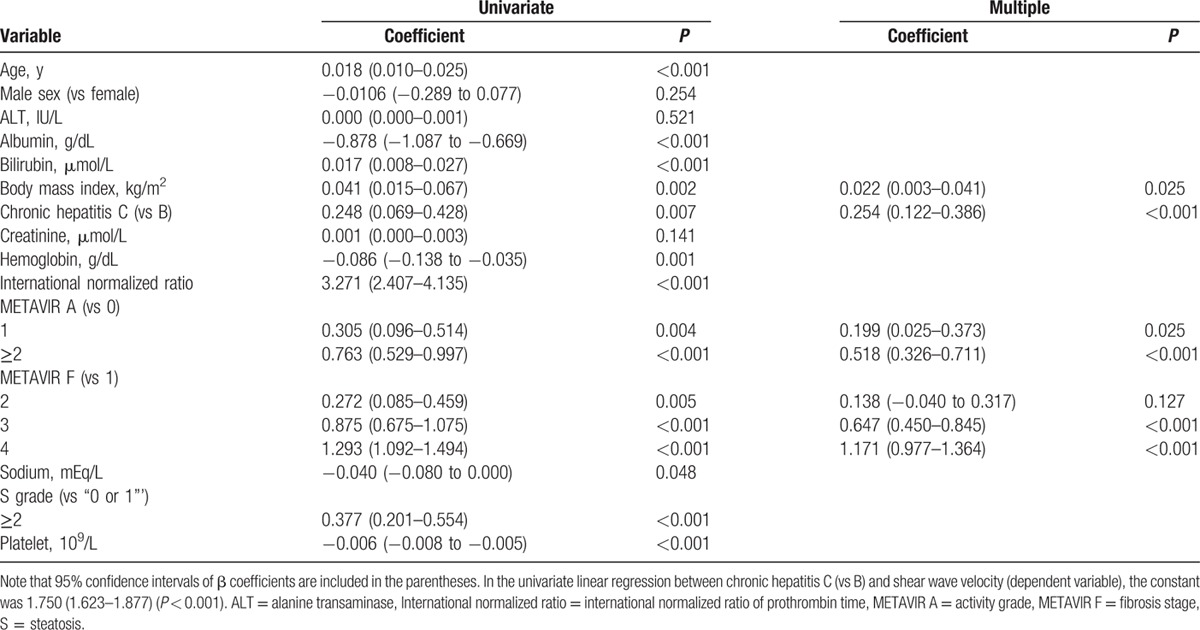
Multiple regression analyses for the shear wave velocity (m/s).

## 4 Discussion

Collagen morphometrics can linearly quantify the extent of liver F in either an area proportion–based or pixel proportion–based manner. Despite the relative operator dependence of nonautomated thresholding for the separate determination of the positivity of the fractal areas and pixels, extracellular matrix collagen morphometry is superior to conventional F stages, which are categorized by staging the architectural changes of liver parenchymal F, rather than quantifying the true F amount. Therefore, collagen morphometry can serve as a sensitive modality for chronologically delineating the bidirectional liver fibrogenesis model in either clinical or research settings.

In the present study, the collagen morphometry refined the F quantification by excluding collagen portions that were irrelevant to the viral hepatitis process. These excluded collagen portions were typically confined to the vascular or biliary walls. Therefore, the nonautomated area proportion–based collagen morphometry is expected to be promising and consistent with collagen-content assays. Moreover, the morphometry was implemented through consensus between a pathologist and hepatologists to ensure precision and accuracy. Future studies may investigate potential inter- and intraobserver variations in the total CPA.

Compared with trichrome and reticulin stainings, the interpretation of pixel- or area-based positivity with picrosirius red collagen staining is a superior method for quantifying the extent of F because of the affinity of picrosirius red with types 1 and 3 of the liver collagens.^[15,29]^ To correlate with several of the serum models of F evaluation, the CPAs using picrosirius red were shown to be significantly more accurate than CPAs using trichrome. However, in the present study, consecutive liver tissue sections were still concomitantly stained using trichrome and reticulin to assist in nonautomated thresholding.

Similar to the variations in LSM, the CPA measurement results likewise varied between the cohorts.^[15]^ The collagen content in liver can be dependent on pathogenesis including distinct viral etiologies. However, the attributions of viral hepatitis etiologies to CPA have seldom been estimated. Despite the potential confounding factors that must be considered, the simple between-group comparisons (Fig. 3) and multiple regressions (Table 3) conducted in this study characterized viral hepatitis etiology as one of the significant explanatory factors of CPA. The significance of viral hepatitis etiology necessitates the indications to stratify the patients studied either in clinical or research settings into CHB and CHC subgroups separately when evaluating the diagnostic performances (AUC, sensitivity, specificity, etc.) of CPA and SWV and applying the cutoff values dichotomizing the conventional liver F stages. Nonetheless, the conventional F staging based on architecture alone may not differ between the viral hepatitis etiologies.

Moreover, F stage-stratified incidence of HCC reported using the person-years method can be compared between 2 published reports on separate Japanese populations diagnosed with untreated CHB^[30]^ or CHC.^[31]^ Apparently, the F stage-stratified HCC incidences were overall estimated to be higher in the CHC than in the CHB groups. Within the F stage of METAVIR F4 alone, the annual incidence rate of HCC was up to 7.88% in the CHC group,^[31]^ in contrast to 4.82% in the CHB group.^[30]^ This type of comparison has been scant to date but can be partially explained by our current F stage-stratified comparisons revealing the higher CPAs in the CHC than in the CHB groups.

Both CPA and SWV are promising modalities for liver F quantification (Table 2). However, both the CPA and SWV measurements tended to be higher in the CHC group than in the CHB group (Fig. 3). These findings may be explained mainly by the growth of PB area proportions during the fibrogenesis process from portal F (METAVIR F1) to cirrhosis (F4). Inversely, significant decreases in area proportions were labeled as PSPA with significantly broadening PBPA. At the stage of cirrhosis, PBPA approximated the total CPA, and the PSPA became relatively scant. Therefore, PBPA more validly reflected F stages and SWV than did PSPA. In our observations, the CHC group exhibited broader septa and spurs on histology than did the CHB group. The global hepatic stellate cell activation index, measured by the immunoreactivity of the surrogate α-smooth muscle actin, was also found by Sturm et al^[17]^ to be higher in the CHC group than in the CHB group. However, this activation index was estimated to be predominately correlated with the defined PS F PA, which was estimated as the proportion between the PS F area and the defined parenchymal area.

The present study had several relevant limitations. First, utilization of the CPAs was intended to enhance the conventional liver F staging system; however, analysis of the CPAs relied on this staging system. Second, the statistical comparisons between the CHB and CHC groups were performed using the nonparametric Mann–Whitney *U* test alone. The numbers of cases in the present study were limited, making it difficult to make comparisons through further substratification by grades of METAVIR A and S to explain the SWVs. Although the grades for necroinflammation were observed to be higher overall in the CHC group than in the CHB group, multiple regression analysis revealed an insignificant correlation between METAVIR A and CPA. Third, despite potential operator dependence, the nonautomated manual approach provides superior identification of fractal areas that must be subtracted—such as lumens, defects, and artifacts—than do automated methods, which require calibration of automated thresholdings, magnifications, and resolutions. The nonautomated quantification of live F remains one of the most practical and accessible approaches worldwide for the study of the invaluable resources of liver tissue sections. Fourth, the PS proportions in the present study were generally lower than those acquired by Sandrini et al^[18]^ across lower F stages (METAVIR F0, F1, and F2), but were comparable to those obtained by Sturm et al^[17]^ at the lower F stages. One of the probable reasons for this may be that distinct quantification methodologies were applied. When the PB proportion areas occupied an increasing amount of the entire section area from MEATVIR F1 through F4, the proportions of PSPA observed through the nonautomated approach became increasingly limited. Future studies may utilize immunostaining for the various types of collagen^[32]^ to provide more accurate and precise quantification of the PSPA or Disse space F than that found in the present study. Fifth, although the relevant confounding factors such as age and sex have been statistically adjusted for, eventually they exhibited less-significant correlations than viral hepatitis etiology and F stage to explain the CPA and SWV, respectively (Tables 3 and 4). Besides, age does not significantly affect the original results indicating the effects of viral hepatitis etiologies on CPAs (refer to figure, Supplemental Digital Content which illustrates the box plots for CPA stratified by age, F stages, and viral hepatitis etiologies). The present case numbers were limited to further facilitate the analysis by concomitantly stratifying by both the F stages and multiple age categories.

In conclusion, both the F stage-stratified CPAs and SWVs tended to be higher in the CHC group than in the CHB group. The type of viral hepatitis significantly affected both the CPA and SWV values. Therefore, this variable must to be taken into account when quantifying liver F using CPA or SWV. The PBPAs more closely correlated with F stages and SWV than did PSPAs. Both CPA and SWV are promising diagnostic solutions for liver F quantification.

## Supplementary Material

Supplemental Digital Content
