# Supplementary material for: Comparison of collagen proportionate areas in liver fibrosis quantification between chronic hepatitis B and C
Source: Medicine (Baltimore). 2016 Sep 2;95(35):e4736. doi: 10.1097/MD.0000000000004736 (PMC5008601; doi:10.1097/MD.0000000000004736)
Supplement: Supplemental Digital Content [file medi-95-e4736-s001.pdf]

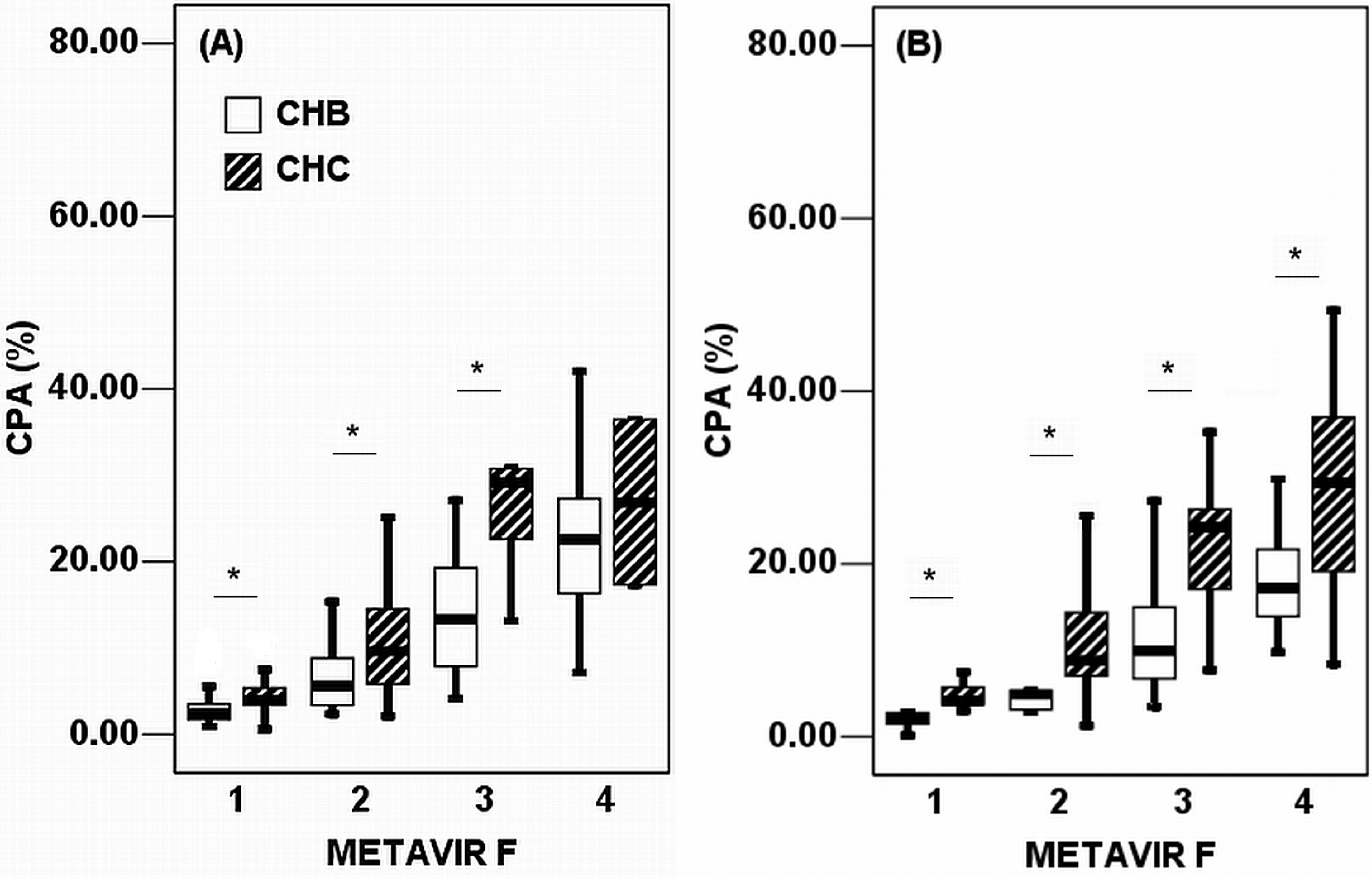

Chen SH et al. Comparison of Collagen Proportionate Areas in Liver Fibrosis Quantification Between Chronic Hepatitis B and C
